# Quantification of urinary TIMP-2 and IGFBP-7: an adequate diagnostic test to predict acute kidney injury after cardiac surgery?

**DOI:** 10.1186/s13054-014-0717-4

**Published:** 2015-01-06

**Authors:** Anna J Wetz, Eva M Richardt, Saskia Wand, Nils Kunze, Hanna Schotola, Michael Quintel, Anselm Bräuer, Onnen Moerer

**Affiliations:** Department of Anaesthesiology, Emergency and Intensive Care Medicine, University of Goettingen, Robert-Koch-Strasse 40, 37075 Goettingen, Germany

## Abstract

**Introduction:**

Postoperative acute kidney injury (AKI) is a frequently observed complication after on-pump cardiac surgery (CS) and is associated with adverse patient outcomes. Early identification of patients at risk is essential for the prevention of AKI after CS. In this study, we analysed whether urinary tissue inhibitor of metalloproteinase 2 (TIMP-2) combined with urine insulin-like growth factor binding protein 7 (IGFBP-7) ([TIMP-2] × [IGFBP-7]) is an adequate diagnostic test to identify early AKI after on-pump CS.

**Methods:**

In 42 patients undergoing coronary artery bypass graft surgery, we surveyed individual risk factors for AKI and defined AKI by applying the Kidney Disease: Improving Global Outcomes (KDIGO) classification during the day of surgery and the following 2 days after surgery. Concentrations of urinary TIMP-2 multiplied by IGFBP-7 were recorded at four time points: at baseline pre-surgery, at the end of surgery, 4 hours after cardiopulmonary bypass (CPB) and at 8:00 am on the first postoperative day.

**Results:**

In total, 38% of the patients experienced AKI. The results showed a median baseline [TIMP-2] × [IGFBP-7] concentration of 0.3 (ng/ml)^2^/1,000, decreasing at the end of surgery and then increasing at the next measurement point 4 hours after CPB and further on the first postoperative day. On the first postoperative day, patients with AKI had significantly higher concentrations of [TIMP-2] × [IGFBP-7]. On the day of surgery, the concentration did not significantly differ between patients classified as KDIGO 0 or KDIGO 1 or 2. Previously published cutoff points of 0.3 and 2 were not confirmed in our study cohort.

**Conclusion:**

[TIMP-2] × [IGFBP-7] concentration can be used as a diagnostic test to identify patients at increased risk of AKI after CS on the first postoperative day. At earlier time points, no significant difference in [TIMP-2] × [IGFBP-7] concentration was found between patients classified as KDIGO 0 or KDIGO 1 or 2.

**Trial registration:**

German Clinical Trials Register (DRKS) DRKS00005457. Registered 26 November 2013.

## Introduction

Acute kidney injury (AKI) is one of the most common organ dysfunctions in critically ill patients [1]. It is frequently observed in patients after cardiac surgery (CS) involving cardiopulmonary bypass (CPB) [2].

The medical and economic consequences of AKI are substantial [3]. AKI requiring renal replacement therapy (RRT) has been reported in 5% of all patients after CS; 8% to 15% present with increased serum creatinine of >1.0 mg/dl; and a discrete increase in creatinine of 25% from baseline is found in 50% [4]. The postoperative mortality rate after CPB lies between 2% and 8%, but it increases exponentially up to 60% in patients requiring RRT [5,6].

In order to apply preventive and therapeutic measures, it is essential to estimate the individual risk and severity of AKI early. Several biomarkers for predicting AKI were identified during the last decade, but none reached an acceptable level of suitability or precision [7]. Recently, a test that quantifies urinary tissue inhibitor of metalloproteinase 2 (TIMP-2) and urinary insulin-like growth factor binding protein 7 (IGFBP-7) has been introduced [8]. TIMP-2 and IGFBP-7 are involved in G_1_ cell cycle arrest of renal tubular cells during the early period of cell injury caused by ischaemic or inflammatory processes which interrupt the integrity and division of cells with damaged DNA until DNA repair is accomplished [9-12].

Kashani *et al*. demonstrated that multiplication of quantified TIMP-2 and IGFBP-7 results in accurate prediction of AKI risk (area under the receiver operating characteristic (ROC) curve (AUC), 0.80) [8]. In the same context, Meersch and colleagues confirmed that urinary [TIMP-2] × [IGFBP-7] concentration is a sensitive and specific biomarker to identify AKI following CS [13].

Before broader routine clinical implementation, a thorough analysis of the diagnostic strength of this method to accurately predict AKI should be repeatedly shown in clinical trials. Accordingly, the aim of this study was to clarify the question whether quantification of TIMP-2 and IGFBP-7 is an adequate diagnostic test for detecting AKI after CS involving CPB.

## Material and methods

We included patients admitted to our ICU at the Department of Anesthesiology and Intensive Care, University Hospital of Goettingen, Germany, after they had undergone CS with the use of CPB. The trial was approved by the local ethics committee of the Medical University of Goettingen (Ethik-Kommission der Universitätsmedizin Göttingen; registration number 17/2/13) and is registered in the German Clinical Trials Register (DRKS), national trial registration number DRKS00005457, http://apps.who.int/trialsearch/ (registered 26 November 2013)). It was planned as a substudy of a clinical trial with the aim of analysing the impact of haemolysis during CPB.

The recruitment period spread from August 2013 to February 2014. The inclusion criteria were that the patient had to give informed consent and had to have undergone (elective or nonelective) coronary artery bypass graft (CABG) surgery with the use of CPB. The exclusion criteria were age younger than 18 years, additional simultaneous valvular or vascular non–coronary artery surgery, preexistent chronic renal failure requiring RRT, CS planned without the use of CPB, heart transplant patients, preoperative cardiac failure requiring extracorporal membrane oxygenation, participation in conflicting studies and refusal to participate in the study.

The following biometrical characteristics, comorbidities and surgical factors were surveyed: sex, age and body mass index (BMI), left ventricular ejection fraction (EF), New York Heart Association congestive heart failure (CHF) classification, chronic obstructive pulmonary disease (COPD), insulin-dependent diabetes mellitus (IDDM), use of a preoperative intra-aortic balloon pump and preoperative concentration of serum creatinine. As surgery-related factors, we reported CABG as primary, redo, elective or emergency surgery; duration of CPB and aortic cross-clamping (ACC) time; and blood and coagulation products received. Individual risk stratification was accomplished by determining the clinical predictive score according to the method of Thakar and colleagues [6].

Urine measurements were performed at four time points to evaluate the earliest possible time frame within an everyday routine clinical setting to diagnose AKI using a commercially available test (NephroCheck test, Astute140 Meter; Astute Medical, San Diego, CA, USA). Measurements were performed at the following time points:

1. Baseline: before surgery and directly after induction of anaesthesia and urinary catheterization
2. End of surgery
3. 4 hours after arrest of CPB
4. 8:00 am on the first postoperative day (1 day after surgery)

To apply the test, we used several commercially available supplies (NephroCheck test slide with enclosed test stripes, buffer solution, test conjugate, quality control kit, 100-μl pipette). Urine samples were taken from urinary catheter tubes under sterile conditions and processed immediately after withdrawal. They were stored at room temperature for a maximum of 2.5 hours before being processed for testing (according to the manufacturer’s recommended maximum of 4 hours).

Buffer solution (100 μl) was mixed with 100 μl of the urine sample and added to the conjugate vial. A quantity of 100 μl of the mix was applied to the cone of the test slide and spread on the test stripe. After incubation, an immunoassay was performed using the Astute140 Meter, and after 20 minutes the result of the multiplied concentration of the biomarkers ([TIMP-2] × [IDFBP-7]) was accomplished. Threshold results were displayed as >0.02 and regarded as 0.02 for statistical analyses.

Postoperatively, the definition and graduation of AKI were determined by applying the Kidney Disease: Improving Global Outcomes (KDIGO) classification criteria [14]. Daily KDIGO stage was determined on the basis of diuresis rate and serum creatinine concentration recorded on the day of surgery and on the following 2 postoperative days. We considered the maximum KDIGO stage achieved by each patient during the postoperative observation period. Patients were classified as KDIGO 0, 1 or 2. The cohort was analysed in groups of KDIGO stage 0 and KDIGO stage >0 with subgroups of KDIGO 1 and KDIGO 2.

The baseline characteristics of the patients were described using median (25th and 75th percentiles) for continuous variables, as all data were non-normally distributed, and number (percentage) for categorical variables. All analyses were completed with Statistica (Version 10, 1984–2011; StatSoft, Tulsa, OK, USA) and SPSS (IBM SPSS, Chicago, IL, USA) software and included testing of distribution with the Kolmogorov-Smirnov test, Mann–Whitney *U* test, χ^2^ test and Fisher’s exact test, as well as logistic regression. Previously published cutoff points were evaluated by calculating sensitivity and specificity after applying them to our patient cohort. ROC analysis was used to determine optimal cutoff points with combined maximized sensitivity and specificity (by Youden index). Significance testing was two-sided with a significance level of 0.05. A *P*-value <0.05 was considered statistically significant.

## Results

Forty-three patients were eligible for the study. One patient died only few hours after surgery, during the day of surgery and was excluded from the study. In one patient, the second and third measurement time points were identical due to prolonged surgery duration after CPB termination, so only one urine sample was investigated for that patient.

The study population consisted of 29 men (69%) and 13 women (31%) with a median age of 72 years (interquartile range, 65 to 76) and a mean BMI of 29.6 kg/m^2^ (Table 1). All but one patient underwent scheduled elective CABG surgery, and none of the patients had undergone CS before. The median length of hospital stay was 12 days. Two patients (4.8%) died postoperatively. The incidence of the preoperative risk factors COPD, IDDM and EF <35% varied between 10% and 33%. Serum creatinine ≥1.2 mg/dl was recorded in 40% of the study cohort (Table 1). According to the Thakar score risk stratification for postoperative AKI (median, 2; range 0 to 6), 57% (*n* = 24) patients were classified as low-risk (0 to 2 points), 40% (*n* = 17) as moderate-risk (3 to 5 points) and 3% (*n* = 1) had 6 score points and were thus at high risk (Table 2). Score points were not significantly higher in the KDIGO 1 or 2 group than in the KDIGO 0 group.

**Table 1:** **Baseline characteristics of the study cohort** ^**a**^

	**All**			**KDIGO 0**			**KDIGO 1 or 2**			
**Variable**	***n*** **= 42**	**%**	**Median (IQR)**	***n*** **= 26**	**%**	**Median (IQR)**	***n*** **= 16**	**%**	**Median (IQR)**	***P*** **-value**
Elective surgery	41	97.6		26	100		15	93.8		0.38
EF <35%	4	9.5		1	3.9		3	18.8		0.15
Preoperative creatinine ≥1.2 mg/dl	26	61.9		8	30.8		9	56.3		0.1
CHF	18	42.9		14	53.9		4	25		0.11
COPD	14	33.3		10	38.5		4	25		0.51
IDDM	10	23.8		6	23.1		4	25		1
Age, yr			72 (65 to 76)			66.5 (61 to 73)			75 (72 to 81)	0.04
BMI, kg/m^2^			29.6 (25.7 to 33.1)			30.1 (26.1 to 33.2)			29.1 (25.7 to 33.1)	0.73
CPB time, min			126.5 (100 to 154)			115.5 (97 to 147)			134.5 (111 to 176.5)	0.17
ACC time, min			75.5 (67 to 103)			70.5 (61–86)			77 (71.5 to 115)	0.12
pRBCs, U			1 (0 to 2)			1 (0 to 2)			0.5 (0 to 3)	0.98
Hospital LOS, days			12 (3 to 33)			12 (9 to 14)			11.5 (9 to 17.5)	0.58
ICU LOS, days			2.5 (1 to 5)			1 (1 to 4)			4.5 (2.5 to 5.5)	0.02

^a^ACC, Aortic cross-clamping; BMI, Body mass index; CHF, Congestive heart failure; COPD, Chronic obstructive pulmonary disease; CPB, Cardiopulmonary bypass; EF, Left ventricular ejection fraction; ICU, Intensive Care Unit; IDDM, Insulin-dependent diabetes mellitus; LOS, Length of stay; pRBCs, Packed red blood cells. Descriptive statistics are displayed as the distribution of the potential risk factors of acute kidney injury separately for the total cohort and for the subcohorts of Kidney Disease: Improving Global Outcomes (KDIGO) stage 0 and KDIGO stage 1 or 2. In the upper rows, categorical preoperative variables are displayed as numbers and ratios of each cohort; in the lower rows, continuous and intraoperative variables are presented as median and interquartile range (IQR, 25th to 75th percentiles), BMI as mean and 95% CI.

**Table 2:** **Distribution of Thakar score points** ^**a**^

**Thakar score** ^**b**^	**All,** ***N*** **= 42**		**KDIGO 0,** ***n*** **= 26**		**KDIGO 1 or 2,** ***n*** **= 16**	
	***n***	**%**	***n***	**%**	***n***	**%**
0	7	16.7	4	15.4	3	18.8
1	11	26.2	9	34.6	2	12.5
2	6	14.3	4	15.4	2	12.5
3	6	14.3	2	7.7	4	25
4	9	21.4	4	15.4	5	31.3
5	2	4.8	2	7.7	0	0
6	1	2.4	1	4.0	0	0

^a^KDIGO, Kidney Disease: Improving Global Outcomes. ^b^Thakar *et al*. [6]. In the absence of risk factors, 0 points are given; a maximum score of 17 points is possible. The study cohort consisted of patients with 0 to 6 Thakar points, with most receiving 0 to 4 points.

Univariate analysis of sex, preoperative serum creatinine level, COPD, IDDM, EF <35%, CHF, elective or nonelective surgery revealed no significant differences between patients with versus without AKI.

Median CPB time was 127 minutes, and median ACC-time was 76 minutes, both of which were longer, but not significantly so, in the KDIGO 1 or 2 group than in the KDIGO 0 group. Patients received in median one red blood cell transfusion; other products were rarely given. No significant differences in distribution were observed for univariate analysis of BMI, blood and coagulation products or length of hospital stay (Table 1). However, patients in the AKI group were found to be significantly older (75 years vs. 66.5 years) and to have stayed longer in the Intensive Care Unit (4.5 days vs. 1 day).

Sixteen patients (38%) experienced AKI within 60 hours postoperatively, whereas twenty-six patients (62%) had no AKI. Thirteen patients (31%) were classified as KDIGO 1 and three patients (7%) as KDIGO 2. KDIGO 1 was achieved due to serum creatinine rise in seven patients, and six patients had primarily oliguria. KDIGO 2 was classified twice on the basis of oliguria, and one patient showed an increase of serum creatinine (Table 3).

**Table 3:** **Analysis of acute kidney injury** ^**a**^

	***n***	**%**	**Creatinine criterion met,** ***n***	**Diuresis criterion met,** ***n***
All	42	100	8	8
KDIGO 0	26	62	–	–
KDIGO 1	13	31	7	6
KDIGO 2	3	7	1	2
KDIGO 3	0	0	–	–

^a^Thirty-eight percent of the patients had acute kidney injury (AKI), and sixty-two percent were classified as Kidney Disease: Improving Global Outcomes (KDIGO) stage 0. Numbers and ratios of patients separated into each KDIGO stage are displayed. The KDIGO stage was determined on the basis of the creatinine or diuresis criterion. Eight patients were defined as patients with AKI primarily on the basis of increased serum creatinine concentration, and eight patients first reached the threshold of a low diuresis rate.

The results of the concentration of ([TIMP-2] × [IGFBP-7]) (ng/ml)^2^/1,000 revealed a baseline median concentration of 0.3 (ng/ml)^2^/1,000, decreasing at the end of surgery to 0.08 (ng/ml)^2^/1,000 and then increasing at the next measurement point 4 hours after CPB and further on the first postoperative day.

**Figure 1:**
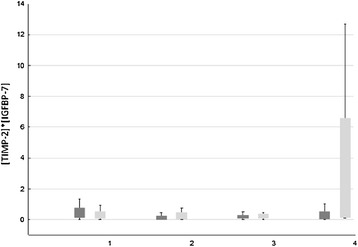
**Boxplots grouped by Kidney Disease: improving global outcomes stage (0 vs. 1 or 2) and time.** The measurement of urinary tissue inhibitor of metalloproteinase 2 (TIMP-2) combined with urinary insulin-like growth factor binding protein 7 (IGFBP-7) using the diagnostic test in 42 cardiac surgery patients revealed that the test was able to discriminate between patients without (dark grey) acute kidney injury (AKI) and those with (light grey) AKI on the first postoperative day (area under the receiver operating characteristic curve, 0.706). The *x*-axis is measurement time points 1 through 4. The *y*-axis is the combined concentration of [TIMP-2] × [IGFBP-7] (ng/ml)^2^/1,000.

On the day of surgery, the concentration of ([TIMP-2] × [IGFBP-7]) (ng/ml)^2^/1,000 did not significantly differ between KDIGO 0 and KDIGO 1 or 2. At the first postoperative day, the median [TIMP-2] × [IGFBP-7] concentration of patients without AKI was 0.28 (ng/ml)^2^/1,000, whereas patients with AKI had a significantly higher [TIMP-2] × [IGFBP-7] concentration of 0.79 (ng/ml)^2^/1,000 (Figure 1 and Table 4).

**Table 4:** **Characteristics of combined urinary concentration of tissue inhibitor of metalloproteinase 2 combined with insulin-like growth factor binding protein 7** ^**a**^

	**All**		**KDIGO 0**		**KDIGO 1 or 2**		
	**Median**	**IQR**	**Median**	**IQR**	**Median**	**IQR**	***P*** **-value** ^**b**^
Baseline	0.3	0.13 to 0.59	0.33	0.13 to 0.73	0.27	0.12 to 0.525	0.6
End of surgery	0.08	0.03 to 0.28	0.07	0.03 to 0.23	0.07	0.05 to 0.48	0.17
4 hr after CPB	0.17	0.09 to 0.31	0.16	0.09 to 0.3	0.18	0.085 to 0.37	0.91
1 day postoperatively	0.28	0.11 to 0.95	0.28	0.08 to 0.52	0.79	0.15 to 6.56	0.03

^a^CPB, Cardiopulmonary bypass; IQR, Interquartile range. The median concentrations of urinary tissue inhibitor of metalloproteinase 2 (TIMP-2) combined with urine insulin-like growth factor binding protein 7 (IGFBP-7) ([TIMP-2] × [IGFBP-7]) (ng/ml)^2^/1,000 at four measurement time points sorted by Kidney Disease: Improving Global Outcomes (KDIGO) groups are displayed. [TIMP-2] × [IGFBP-7] concentration first decreased from baseline after surgery and then increased 4 hours after the arrest of CPB and increased further on the first postoperative day. On the day of surgery (measurement points 1, 2 and 3), no significant difference was found between patients with acute kidney injury (AKI) (KDIGO 1 or 2) and those without AKI (KDIGO 0). On the first postoperative day, patients with AKI had significantly higher concentrations of urinary [TIMP-2] × [IGFBP-7]. ^b^ *P* < 0.05 by Mann–Whitney *U* test was considered statistically significant.

Applying previously published cutoff concentrations to our results (first postoperative day) revealed a sensitivity of 53% and a specificity of 54% for the cutoff point of 0.3. The cutoff point of 2 led to a sensitivity of 33% and a specificity of 100% (Table 5).

**Table 5:** **Analysis of applying previously published cutoff points**

	**Cutoff**	**Sensitivity**	**Specificity**
End of surgery	0.3	0.36	0.84
	2	0.07	0.96
1 day after surgery	0.3	0.53	0.54
	2	0.33	1

The sensitivity and specificity of applied previously published cutoff points of 0.3 and 2 are shown. The highest sensitivity was found at the cutoff point of 0.3 on the first postoperative day; the highest specificity was found with a cutoff point of 2 on the first postoperative day.

By ROC analysis, we determined optimized cutoff points of the multiplied biomarker concentration of the second and last measurement time points. For the baseline and third (4 hours after CPB) measurement time points, we found AUCs of 0.451 and 0.512, respectively, so no cutoff point was built. For the second measurement time point, a cutoff concentration of 0.41 (36% sensitivity, 92% specificity) was determined and the last measurement time point defined a cutoff point of 1.065 (47% sensitivity, 96% specificity). The AUCs were 0.634 at the second measurement time point and 0.706 at the last measurement time point. The diagnostic test was able to discriminate between patients with and those without AKI on the first postoperative day (AUC, 0.706; *P* = 0.029).

## Discussion

In this study, we analysed the precision and discriminative ability of a new set of biomarkers to predict AKI after CS with CPB. We selected three postoperative measurement points (end of surgery, 4 hours after the arrest of CBP and at 8:00 am on the first postoperative day) to estimate the earliest possible time frame to predict or diagnose AKI. On the day of surgery, urinary [TIMP-2] × [IGFBP-7] concentration did not significantly differ between patients with versus those without AKI. On the first postoperative day, the median concentration of [TIMP-2] × [IGFBP-7] was significantly higher in patients with AKI. This implies that the diagnostic test is able to predict AKI and discriminate between patients with versus those without AKI on the first postoperative day, but that it fails to do so on the day of surgery.

Our results indicate that measurement time points on the day of surgery were selected too early and could not predict AKI, because no increase from baseline was observed and no significant differences were found between patients with versus those without AKI. We were not able to identify AKI risk 4 hours after CPB as Meersch *et al*. did [13]. The reason for this difference may lie in different patients and surgical settings, as we included only patients undergoing CABG surgery and our study cohort was not limited to high-risk AKI patients. On the first postoperative day, the test succeeded in AKI prediction.

Our study presents a new aspect of diagnostic prediction by analysing the concentrations of ([TIMP-2] × [IGFBP-7]) (ng/ml)^2^/1,000 in all patients undergoing CABG surgery and not only in patients at high risk for AKI. The fact that we did not focus on only high-risk patients, but included all patients undergoing CABG surgery, is reflected in the absence of risk factors for AKI (established according to the scoring method of Thakar *et al*. [6]) in 16.7% of the study cohort. The previously published trials included only patients with at least one defined risk factor [8] or patients with 6 or more Thakar points. In contrast, our study included only 43% of patients with more than 2 Thakar points, but 57% of patients had ≤2 points. Because an AKI incidence of 38% still can be found in patients with 0 to 4 Thakar points, we considered it important to determine whether the diagnostic test is also applicable in this lower risk cohort.

Compared to surgical procedures with valve or combined surgery, CABG-only surgery requires shorter CBP and ACC time, assuming lesser injury to the kidneys. So, it is possible that the AKI of lesser severity reached in our patient population was due to different surgical conditions and not of a time frame within which the biomarker concentration rises to a significant level at early measurement time points. The risk of injury to the kidneys could have been considerably higher in the trial of Meersch and colleagues [13] than in our present study, because they also included patients undergoing valvular and combined surgeries with assumed higher risks of kidney injury due to longer CBP and ACC times than we observed in our patient population (140 and 98 minutes vs. 127 and 78 minutes, respectively). This may have led to the divergent results and may be a reason why the trials of Kashani *et al*. [8] and Meersch *et al*. [13], who included only patients at higher risk for AKI, showed more precise results of the biomarker test.

Because the test was not perfectly applicable to our patients of mainly low-grade kidney injury (KDIGO 1) and Kashani *et al*. proved good results on KDIGO 2 and 3, the question arose wheather the biomarker test might be better applicable for predicting high-grade kidney injury [8].

Previously published cutoff points of 0.3 and 2 [8] could not be confirmed in our study cohort. Applying the low cutoff point resulted in a sensitivity of 53% and a specificity of 54%. The cutoff point of 2 led to a sensitivity of 33% and a specificity of 100%. In contrast, in our present study, we found a cutoff point of 1.1 with an AUC of 0.71 (46.67% sensitivity, 96.15% specificity). The reason for this finding may lie again in different patient cohort composition and different surgical settings, as well as our smaller number of patients, which surely led to weaker results.

Another difference between the studies can be found in the trend of [TIMP-2] × [IGFBP-7] concentration. We observed higher baseline concentrations, with decreasing trends for the following measurement points. This finding does not correspond to the results of the earlier trials. This may be attributable to a different fluid management concept at our department during CS that relies mainly on crystalloid solutions instead of colloids, as well as a low rate of transfusion during CABG surgery.

AKI was determined on the basis of the KDIGO classification system, which provides an objective, comparable, precise definition of AKI and corresponds to current standards [14]. Clinical tools used to detect AKI, such as serum creatinine rise and measurement of urine output, are well established and easy to determine in routine clinical practice. Nevertheless, they can be influenced by various unspecific factors, such as muscle mass and volume status, and do not reflect glomerular filtration or the degree of tubular injury [15-17].

An adequate test would confirm a diagnosis in a timely manner with high discrimination and precision; would be superior to, or at least complement, established clinical markers; and would lead to improvement of therapeutic management and patient outcome. The introduced commercial NephroCheck test of combined urinary TIMP-2 and IGFBP-7 concentrations, which are involved in cell cycle pathways of renal tubular cells [9-12], is easy to perform with a small urine sample; the results are displayed after 20 minutes.

The question whether this diagnostic test is adequate to predict AKI after CS can be answered affirmatively with a trend towards moderate sensitivity and specificity on the first postoperative day. Not only accurate prognostic stratification but also early diagnosis of AKI are essential for optimal medical management [18]. Clinical markers of kidney function often fail to detect AKI at a time when interventions may provide benefit [8]. The observed clinical markers, known for their delayed reaction, increased in 62.5% of patients until the first postoperative day and in 37.5% on the second postoperative day. Because the combined [TIMP-2] × [IGFBP-7] concentration performed poorly on the day of surgery and started to discriminate between patients with and those without AKI risk not prior to, but on, the first postoperative day, the advantage in earlier identification of AKI risk over clinical markers and the value for early anticipation of the immediate risk of AKI [19] remain to be elucidated.

Our results raise the question whether the biomarker product stratifies AKI more precisely than KDIGO criteria do, and therefore the benefit of comparison might be limited. Thus, the severity of AKI might differ between two patient cohorts when both are clinically classified as KDIGO stage 1 or 2, a difference that may be identified and stratified only by biomarkers. Biomarkers may then lead to an expansion of diagnostic criteria for AKI by allowing detection of patients with a subclinical risk of kidney injury when routine diagnostic tools such as serum creatinine and urine output stay unchanged [20,21]. Furthermore, testing of biomarkers sheds light on the individual pathogenesis of AKI [8], enabling differential diagnosis in critically ill patients.

However, the value of successfully predicting AKI at this time point needs further assessment. After investigations into how biomarkers would be utilized by clinicians and into the form of application in clinical practice [20], further trials are needed to clarify the question whether the use of biomarkers leads to consequences in therapeutic management or improvement in clinical outcomes, as recently proposed, by early assessment of prognosis, advanced warning and alerting clinicians [19].

## Conclusion

On the basis of our findings in 42 patients undergoing CABG surgery, we conclude that the recently introduced [TIMP-2] × [IGFBP-7] concentration–based NephroCheck test allows identification of patients at increased AKI risk after CS starting at the first postoperative day, but not during the early postoperative phase. We assume that quantification of [TIMP-2] × [IGFBP-7] concentration may be more suitable in patients undergoing CABG surgery who are at high risk for AKI and may be less precise in patients at low risk for AKI.

## Key messages

- The [TIMP-2] × [IGFBP-7]–based NephroCheck test allowed us to identify patients at increased AKI risk after CS.
- Significant changes of [TIMP-2] × [IGFBP-7] started on the first postoperative day.
- During the early postoperative phase, no significant difference was found between patients with versus those without AKI.

## Abbreviations

ACC: Aortic cross-clamping
AKI: Acute kidney injury
AUC: Area under the receiver operating characteristic curve
BMI: Body mass index
CABG: Coronary artery bypass graft
CHF: Congestive heart failure
COPD: Chronic obstructive pulmonary disease
CPB: Cardiopulmonary bypass
CS: Cardiac surgery
EF: Ejection fraction
ICU: Intensive Care Unit
IDDM: Insulin-dependent diabetes mellitus
IGFBP-7: Insulin-like growth factor binding protein 7
IQR: Interquartile range
KDIGO: Kidney disease: improving global outcomes
LOS: Length of stay
pRBCs: Packed red blood cells
ROC: Receiver operating characteristic curve
RRT: Renal replacement therapy
TIMP-2: Tissue inhibitor of metalloproteinase 2
